# Synergistic interaction between a PDE5 inhibitor (sildenafil) and a new adenosine A_2A_ receptor agonist (LASSBio-1359) improves pulmonary hypertension in rats

**DOI:** 10.1371/journal.pone.0195047

**Published:** 2018-04-20

**Authors:** Allan K. Alencar, Fábio I. Carvalho, Ananssa M. Silva, Sabrina T. Martinez, Jorge A. Calasans-Maia, Carlos M. Fraga, Eliezer J. Barreiro, Gisele Zapata-Sudo, Roberto T. Sudo

**Affiliations:** 1 Programa de Pesquisa em Desenvolvimento de Fármacos, Instituto de Ciências Biomédicas, Universidade Federal do Rio de Janeiro, Rio de Janeiro, Rio de Janeiro, Brazil; 2 Instituto de Química, Universidade Federal Fluminense, Rio de Janeiro, Rio de Janeiro, Brazil; 3 Serviço de Anestesiologia, Hospital Universitário, Universidade Federal do Rio de Janeiro, Rio de Janeiro, Rio de Janeiro, Brazil; Max Delbruck Centrum fur Molekulare Medizin Berlin Buch, GERMANY

## Abstract

**Introduction:**

Pulmonary hypertension (PH) is characterized by enhanced pulmonary vascular resistance, which causes right ventricle (RV) pressure overload and results in right sided heart failure and death. This work investigated the effectiveness of a combined therapy with PDE5 inhibitor (PDE5i) and a new adenosine A2A receptor (A2AR) agonist in mitigating monocrotaline (MCT) induced PH in rats.

**Methods:**

An in vitro isobolographic analysis was performed to identify possible synergistic relaxation effect between sildenafil and LASSBio 1359 in rat pulmonary arteries (PAs). In the in vivo experiments, PH was induced in male Wistar rats by a single intraperitoneal injection of 60 mg/kg MCT. Rats were divided into the following groups: control (saline injection only), MCT + vehicle, MCT + sildenafil, MCT + LASSBio 1359 and MCT + combination of sildenafil and LASSBio 1359. Fourteen days after the MCT injection, rats were treated daily with oral administration of the regimen therapies or vehicle for 14 days. Cardiopulmonary system function and structure were evaluated by echocardiography. RV systolic pressure and PA endothelial function were measured.

**Results:**

Isobolographic analysis showed a synergistic interaction between sildenafil and LASSBio 1359 in rat PAs. Combined therapy with sildenafil and LASSBio 1359 but not monotreatment with low dosages of either sildenafil or LASSBio 1359 ameliorated all of PH related abnormalities in cardiopulmonary function and structure in MCT challenged rats.

**Conclusions:**

The combination of sildenafil and LASSBio 1359 has a synergistic interaction, suggesting that combined use of these pharmacological targets may be an alternative to improve quality of life and outcomes for PH patients.

## Introduction

Pulmonary hypertension (PH) is a vasculopathy of the cardiopulmonary system, which may lead to right ventricle (RV) dysfunction, decompensated heart failure, and significant reduction of life expectancy [1, 2]. A recent study showed that combined targeted therapy for PH significantly lowered the risk of clinical worsening compared with monotherapy [3]. However, many patients still have a poor prognosis when treated with combinations of available clinical drugs. Thus, it is essential to identify innovative therapeutic strategies for PH [3–5].

Pulmonary vessel cells express substantial amounts of PDE5, an enzyme that degrades cGMP [6]. Inhibition of PDE5 has been used as a PH therapy. All three PDE5i approved for the treatment of PH—sildenafil, tadalafil, and vardenafil—induce potent pulmonary vasodilation with a concomitant antiproliferative effect [6, 7].

The A_2A_R is expressed in pulmonary vascular tissue and heart cells [8–10]. Activation of A_2A_Rs was shown to attenuate the cardiopulmonary characteristics in a MCT-induced PH model in male rats [9, 11]. Thus, targeting A_2A_R activation could be an effective and novel therapeutic strategy for mitigating PH characteristics. LASSBio-1359 is a selective A_2A_R agonist with well-characterized vasodilator, antiproliferative, anti-inflammatory, and cardioprotective properties, which are all promoted by A_2A_R activation [11–13]. Furthermore, we recently showed that LASSBio-1359 promoted synergistic relaxation with sildenafil in guinea pig corpus cavernosum [12].

Accordingly, to provide novel insight into PH therapy, we studied the vasoactive properties of a combination of LASSBio-1359 with sildenafil. To date, there is no data concerning the specific *in vitro* and *in vivo* pharmacological effects of this drug combination in the preclinical field. First, we examined the combination of LASSBio-1359 and sildenafil using an isobolographic analysis in rat PAs. Next, the combination of both drugs was administered in rats with MCT-induced PH. We hypothesized that a synergistic interaction between the A_2A_R agonist and PDE5i would modulate PH pathways at multiple sites in the cardiopulmonary system and provide superior efficacy than monotherapy in an MCT-induced PH model.

## Material and methods

### Animals

Male Wistar rats were obtained from the Animal Facility of the Institute of Biomedical Sciences at Universidade Federal do Rio de Janeiro and were maintained at 20 ± 3 °C under a 12 hour light/dark cycle, with water and standard rat chow offered *ad libitum*. Body weight and behavioral state were monitored throughout the protocol indicating the good health condition of all animals. All efforts were made to reduce pain and suffering of the animals, as detailed in this section. The experimental procedures were submitted and approved by the Animal Care and Use Committee at Universidade Federal do Rio de Janeiro (Permit # DFBCICB059).”

### Drugs and reagents

LASSBio-1359 was synthesized by Laboratório de Avaliação e Síntese de Substâncias Bioativas (LASSBio) at Universidade Federal do Rio de Janeiro, Brazil. LASSBio-1359 and sildenafil (Cristália, Itapira, São Paulo, Brazil) were dissolved in DMSO (Cristália, Itapira, São Paulo, Brazil). MCT was synthesized by Laboratório de Química at Universidade Federal Fluminense (Rio de Janeiro, Brazil), and it was dissolved in 1 N HCl, neutralized with 0.5 N NaOH, and diluted with phosphate-buffered saline. Phenylephrine (Phe) and ACh from Sigma-Aldrich (St. Louis, USA) were dissolved in distilled water.

### In vitro isobolographic analyses

Quantitative assessment of the interaction between the relaxation induced by LASSBio-1359 and sildenafil in PA rings from male Wistar rats (220–300 g) was performed using isobolographic analyses. First, the EC_50_ was calculated using previously published methods [9, 11]. A concentration-response curve was then obtained by coaddition of LASSBio-1359 with sildenafil in an organ bath containing PA rings. LASSBio-1359 and sildenafil were added together in fixed ratio (1:3) combinations of fractions of their respective EC_50_ values. Separated PA rings were incubated with (LASSBio-1359 EC_50_ + sildenafil EC_50_)/8, (LASSBio-1359 EC_50_ + sildenafil EC_50_)/4, (LASSBio-1359 EC_50_ + sildenafil EC_50_)/2, (LASSBio-1359 EC_50_ + sildenafil EC_50_) × 1, or (LASSBio-1359 EC_50_ + sildenafil EC_50_) × 2. The EC_50_ of the combinations (EC_50mix_) was determined and used to generate the isobologram plot according to previously published procedures [12, 14–18]. A theoretical additive combination EC_50_ (EC_50add_) was obtained using the following equation:

$$EC_{50add}=\frac{EC_{50}LASSBio-1359}{p1+Rp2}$$

where R is the potency ratio calculated from EC_50 LASSBio-1359_/EC_50 sildenafil_, p1 is the proportion of LASSBio-1359 (25%), and p2 is the proportion of sildenafil (75%) [12, 19].

The additive line was constructed by connecting the EC_50_ of LASSBio-1359 plotted on the ordinate with the EC_50_ of sildenafil plotted on the abscissa. Experimental values that were on or near the additive line were considered to be additive interactions, those that lay below and to the left were considered to be synergistic, and those that lay above and to the right were considered to be subadditive or antagonistic interactions [12, 14–18]. *In vitro* data were analyzed using GraphPad Prism, version 6 (GraphPad, San Diego, USA).

### In vivo model of MCT-induced PH

All experiments were conducted in accordance with the Animal Care and Use Committee at Universidade Federal do Rio de Janeiro (Permit Number: DFBCICB059). Fifty male Wistar rats (220–300 g) were housed at 20 ± 3 °C under a 12-h light/12-h dark cycle with free access to food and water. Rats were randomly divided into two groups: control (C group, n = 6) and MCT (n = 44). Rats in the MCT group were given a single i.p. injection of MCT at 60 mgkg^-1^ body weight to induce PH using previously published methods [9, 11, 20, 21]. Rats in the C group were injected with the same volume of sterile saline.

**Fig 1:**
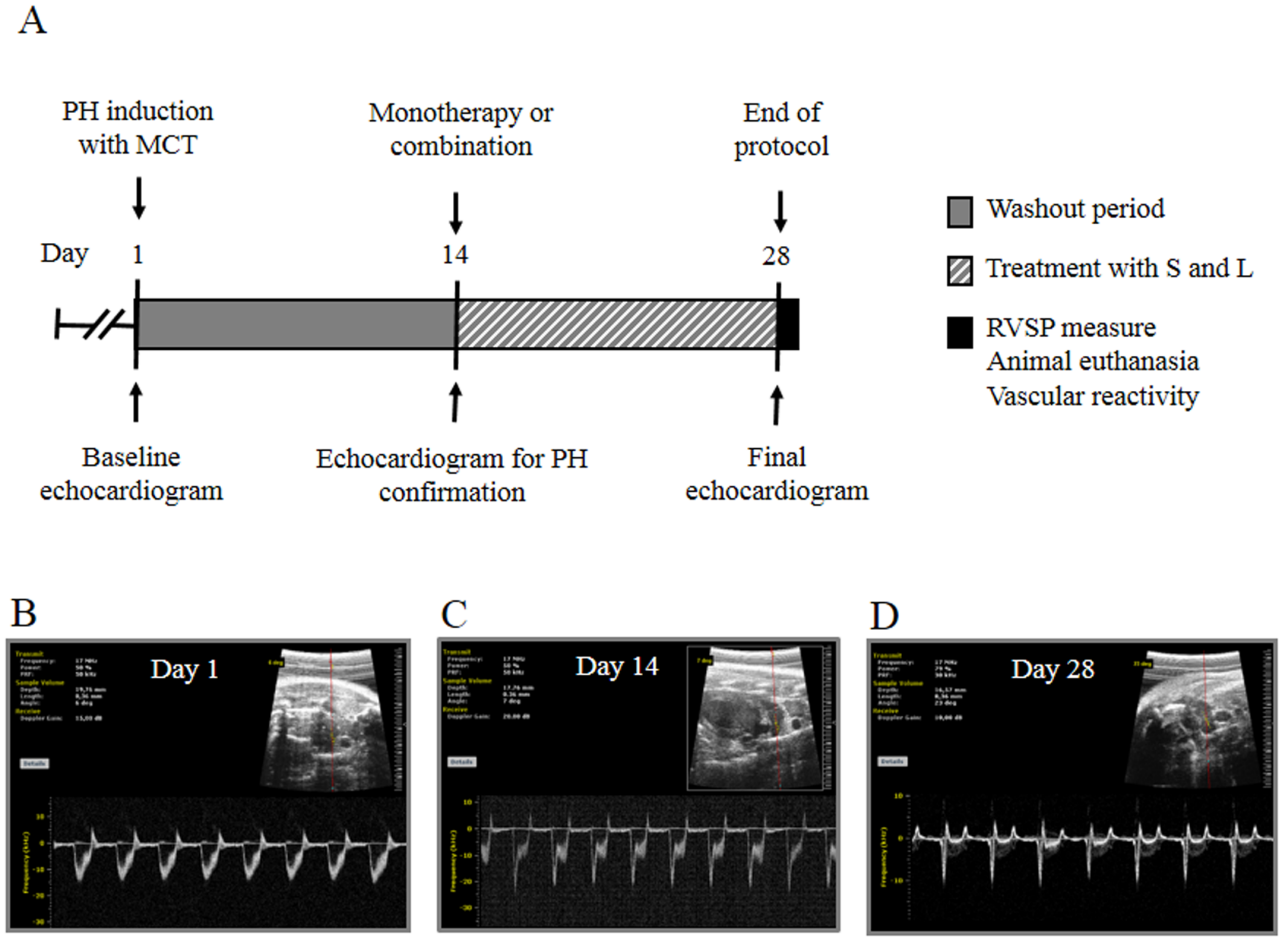
Experimental timeline in days for the monocrotaline (MCT)-induced pulmonary hypertension protocol. **(a)** MCT was injected at a dose of 60 mgkg^-1^ i.p. on day 1. Fourteen days (washout period) after MCT injection, rats were orally treated with sildenafil (S), LASSBio-1359 (L), or both substances for 14 days. On days 1, 14, and 28, echocardiographic examinations were performed to monitor cardiopulmonary system function and structure. On day 28, right ventricle (RV) catheterization was performed to measure RV systolic pressure (RVSP). Following this measurement, the animals were euthanized, the hearts were removed and weighed, and the RV was dissected. Pulmonary arteries (PAs) were rapidly removed for the vascular reactivity study. Representative images of the PA outflow profile are presented for **(b)** day 0, **(c)** day 14, and **(d)** day 28 after MCT injection in an animal from the vehicle (V) group. Note the progressive change in the shape of the PA waveform from day 14 to day 28 after MCT injection, which confirmed the development of PH.

Changes in the PA artery flow profile might be related to the severity of PH, mainly when a triangular flow and a mid-systolic notching are observed [22]. Two weeks after MCT or saline administration, echocardiographic evaluation showed evidence of PH in the MCT-treated rats, including a change in the shape of the PA waveform (Fig 1) in accordance with previous reports [21,22]. After the echocardiographic examination, the MCT-treated rats were divided into six treatment groups (n = 6 per group): 1. 0.2 mL of vehicle (DMSO) which was named as PH group (V group); 2. 34 μmolkg^-1^day^-1^ of sildenafil (S-34); 3. 34 μmolkg^-1^day^-1^ of LASSBio-1359 (L-34); 4. 170 μmolkg^-1^day^-1^ of sildenafil (S-170); 5. 170 μmolkg^-1^day^-1^ of LASSBio-1359 (L-170); 6. sildenafil + LASSBio-1359 (S + L; 34 μmolkg^-1^day^-1^ of each drug). Four of the six rats in the V group died between 14 and 28 days after the MCT injection due to complications caused by the experimental model. Another group of six animals was treated with MCT to replace the deceased rats in the V group, and only four rats survived by the end of protocol (day 28).

### Dose calculations and treatment protocol

Oral administration of LASSBio-1359 at a dose of 170 μmolkg^-1^day^-1^ was shown to attenuate PH in rats challenged with MCT [11]. In this study, the high-dose monotherapies for MCT-injected rats were 170 μmolkg^-1^day^-1^ of sildenafil or LASSBio-1359 to reproduce our previous results. The low-dose monotherapies for MCT-injected rats were 34 μmolkg^-1^day^-1^ (20% of 170 μmolkg^-1^) of sildenafil or LASSBio-1359. In addition, MCT-induced PH rats were coadministered with sildenafil + LASSBio-1359 (34 μmolkg^-1^day^-1^ of each drug); as addressed earlier, to compare the efficacy of the combination with the effects of the low-dose monotherapies. All drugs were administered by oral gavage for 14 days, and the *in vivo* protocol ended on day 28 (Fig 1). Rats were weighed daily, and volumes of solutions were appropriately adjusted.

### Echocardiography

Experimental groups were anesthetized by a 2% isoflurane/oxygen mixture through a nose cone during spontaneous ventilation. Anesthetized spontaneously breathing animals were placed in a shallow left lateral decubitus position. The left hemithorax was shaved and prepared with acoustic coupling gel to increase probe contact. Room temperature was maintained at approximately 25 °C to avoid hypothermia. Cardiac function was assessed by a high-resolution ultrasound imaging system equipped with a RMV-710B transducer with a frequency of 25 MHz and a fixed focal length of 15 mm mounted on an integrated rail system (Vevo 770, Visualsonics, Toronto, Canada). The procedures followed were previously described [21]. Cardiac function was measured before MCT injection (baseline), 14 days after MCT injection, and 28 days after MCT injection (Fig 1).

### RV hemodynamic and hypertrophy measurements

On day 28, 4 hours after the echocardiography examination, rats were anesthetized with ketamine (80 mgkg^-1^, i.p.) and xylazine (15 mgkg^-1^, i.p.; Fig 1). Anesthesia depth was verified by pinching the animal’s paw with forceps. Right-ventricle systolic pressure (RVSP) and RV hypertrophy were measured following procedures published previously [21, 22].

### Evaluation of PA endothelial function in rats with MCT-induced PH

After euthanasia, the branch of PA near to the heart was removed, cleaned of adjacent tissue, and prepared for isometric tension recording. Rings (2 mm) of PA were placed in vertical chambers with modified Tyrode solution (20 mM NaCl, 5.9 mM KCl, 1.2 mM MgCl_2_, 1.2 mM NaH_2_PO_4_, 18mM NaHCO_3_, 1.2 mM CaCl_2_, and 11 mM glucose; pH 7.4) and oxygenated with carbogen gas at 35 ± 0.5 °C. After 2 hours of adaptation at 1.5 g resting tension, preparations were exposed to increasing concentrations of Phe (1 nM– 10 μM). After the plateau of contraction induced by 10 μM, the PA rings were exposure to increasing concentrations of ACh (1 nM– 10 μM) to determine the vasodilation capacity and endothelial function [9, 11].

### Data analysis

Data analysis was performed for all endpoints, and one-way analysis of variance was used to determine the significance of differences among groups. Significance of interactions between groups was determined by Tukey’s post-hoc tests. Pearson correlation was used to test for a relationship between RVSP and ACh-induced maximal relaxation in the PA. Differences between two data points in the *in vitro* studies were compared using unpaired Student’s t test. Differences for all tests were considered significant when the P value was less than 0.05. Analyses were performed using GraphPad Prism, version 6 (GraphPad, San Diego, CA, USA).

## Results

### Sildenafil and LASSBio-1359 induced synergic relaxation in rat PAs

**Fig 2:**
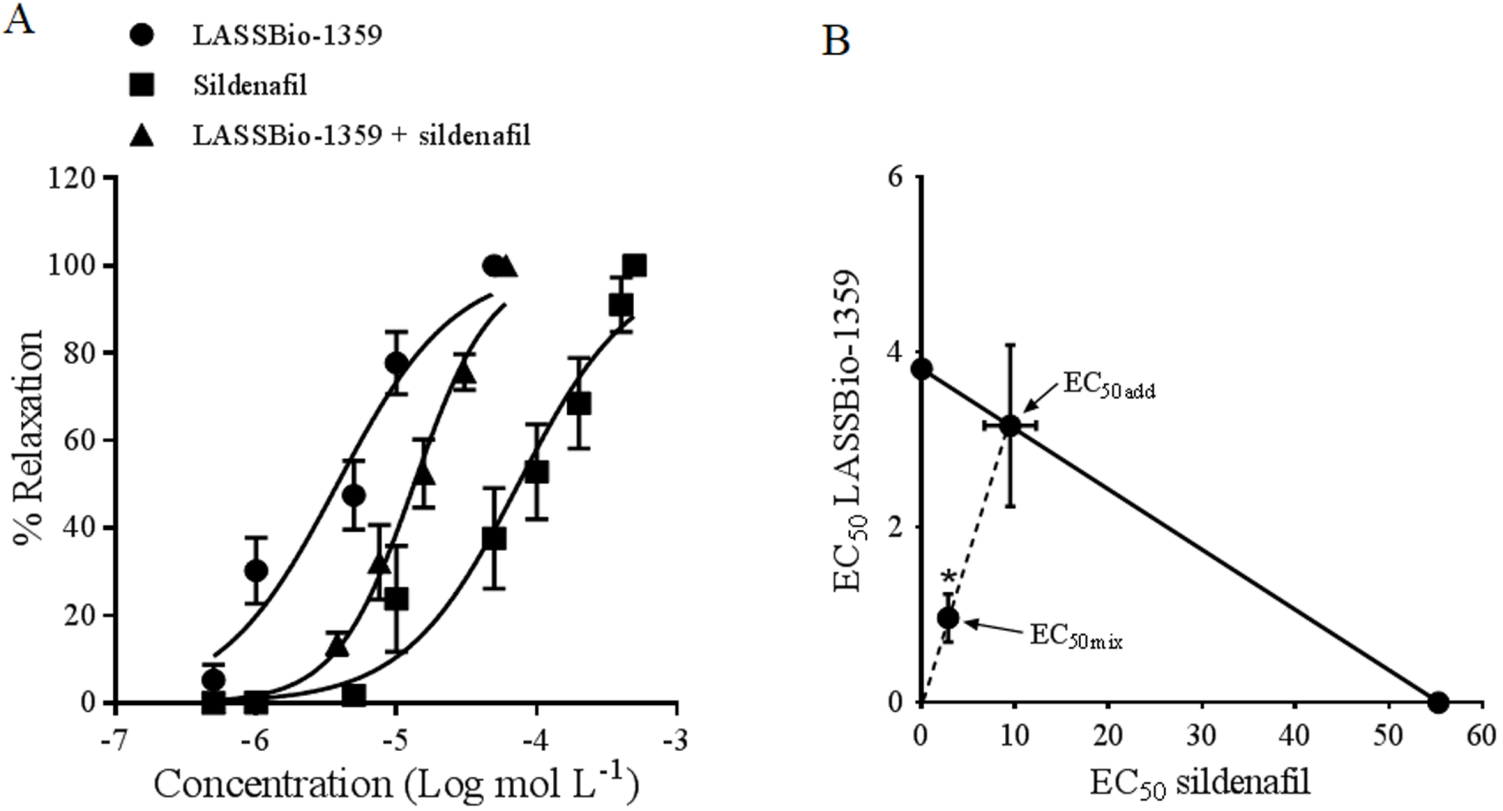
**(a) Relaxation effect of sildenafil, LASSBio-1359, or a combination of these drugs on phenylephrine-induced contraction of isolated pulmonary arteries from male Wistar rats**. Each point represents the mean ± standard error of the mean (SEM) of 6 experiments. **(b)** Isobologram showing the interaction of sildenafil with LASSBio-1359. Note that the experimental EC_50_ is located at the left and below the theoretical EC_50_. A synergistic interaction between sildenafil and LASSBio-1359 was detected using the Student’s *t*-test. *P < 0.05 compared with EC_50add_. EC_50_, concentration of each drug that produces 50% of the maximal relaxation; EC_50mix_, experimental EC_50_ of the drug combination; EC_50add_, theoretical additive combination EC_50_.

Sildenafil caused vasodilation in rat PAs that were precontracted with Phe (10 μM) (Fig 2A). The EC_50_ of sildenafil-induced relaxation (55.3 ± 2.6 μM) was significantly higher than that of LASSBio-1359 (3.81 ± 1.1 μM; Fig 2A). A concentration-response curve of the coadministration of LASSBio-1359 plus sildenafil in combination fractions (1/8, 1/4, 1/2, 1, and 2) was used to calculate the EC_50mix_. The LASSBio-1359 EC_50_ + sildenafil EC_50_ solution (fixed 1:3 EC_50_ ratio) induced relaxation in a concentration-dependent manner, with the maximal effect being reached at fraction 5 (Fig 2A). Vehicle in precontracted vessels did not produce alterations in the contractile response. According to the isobolographic analyses, the calculated theoretical EC_50add_ was 12.6 ± 3.6 μM, which is significantly higher than the experimental EC_50mix_ of 3.7 ± 1.3 μM (Fig 2B). These data suggest a synergistic interaction between sildenafil and LASSBio-1359 in the pulmonary vasculature.

### Effects of treatments on body and heart weights in rats with MCT-induced PH

Animal characteristics are shown in Table 1. The final body weight was significantly lower in the S-170 and L-34 groups compared to the C group animals, which is likely due to the effect of MCT and PH severity. Therefore, we used tibial length (TL), which did not change between the groups due to the protocol (Table 1), to calculate the myocardial hypertrophy indexes. MCT injection significantly increased the ratios of heart weight to TL in comparison to the C group. The Fulton’s index (RV/LV + S) was significantly increased in V group as a sign of RV hypertrophy (Table 1). The changes in heart weight and Fulton’s index observed in MCT-treated rats were reduced when MCT-treated rats were administered high-dose monotherapy with either sildenafil or LASSBio-1359 (170 μmolkg^-1^day^-1^; Table 1). Nevertheless, myocardial hypertrophy was not ameliorated in the S-34 and L-34 groups. In contrast, daily oral administration of a combination of LASSBio-1359 EC_50_ + sildenafil at a dose of 34 μmolkg^-1^day^-1^ for each drug suppressed heart and RV weight changes in rats with MCT-induced PH (Table 1).

**Table 1:** Comparative data on body, heart, and right ventricle weights.

	C	V	S-34	S-170	L-34	L-170	S + L
FBW, g	240.5 ± 6.5	228.5 ± 4.6	227.4 ± 2.5	222.2 ± 4.0*	221.3 ± 2.9*	224.7 ± 1.9	232.0 ± 3.7
TL, mm	36.5 ± 0.2	38.3 ± 0.8	38.3 ± 0.6	38.2 ± 0.5	37.6 ± 0.8	37.1 ± 0.2	37.9 ± 0.4
HW/TL, mgg^-1^	30.0 ± 0.5	41.0 ± 1.8*	38.3 ± 1.0*	31.7 ± 0.4^#^†°	37.9 ± 1.6*	32.2 ± 0.5^#^†°	32.9 ± 1.1^#^†°
RV/LV + S	0.5 ± 0.03	0.9 ± 0.04*	0.9 ± 0.01*	0.7 ± 0.05^#^†°	0.9 ± 0.03*	0.7 ± 0.02^#^†°	0.6 ± 0.05^#^†°

Each value represents the mean ± S.E.M (n = 5 rats per group).

*P < 0.05 compared with control rats,

^#^*P* < 0.05 compared with MCT + vehicle (V) rats,

^†^*P* < 0.05 compared with MCT + sildenafil 34 μMkg^-1^day^-1^ (S-34), and

°*P* < 0.05 compared with MCT + LASSBio-1359 34 μMkg^-1^day^-1^ rats.

C, control; V, MCT + vehicle; S-34, MCT + sildenafil (34 μmolkg^-1^day^-1^); S-170, MCT + sildenafil (170 μmolkg^-1^day^-1^); L-34, MCT + LASSBio-1359 (34 μmolkg^-1^day^-1^); L-170, MCT + LASSBio-1359 (170 μmolkg^-1^day^-1^); S + L, combination of sildenafil and LASSBio-1359 (34 μmolkg^-1^day^-1^ of each drug); FBW, final body weight; HW, heart weight; RV, right ventricle; LV + S, left ventricle plus interventricular septum, TL, tibial length.

### Effects of treatments on PA flow 4 weeks after MCT injection

**Fig 3:**
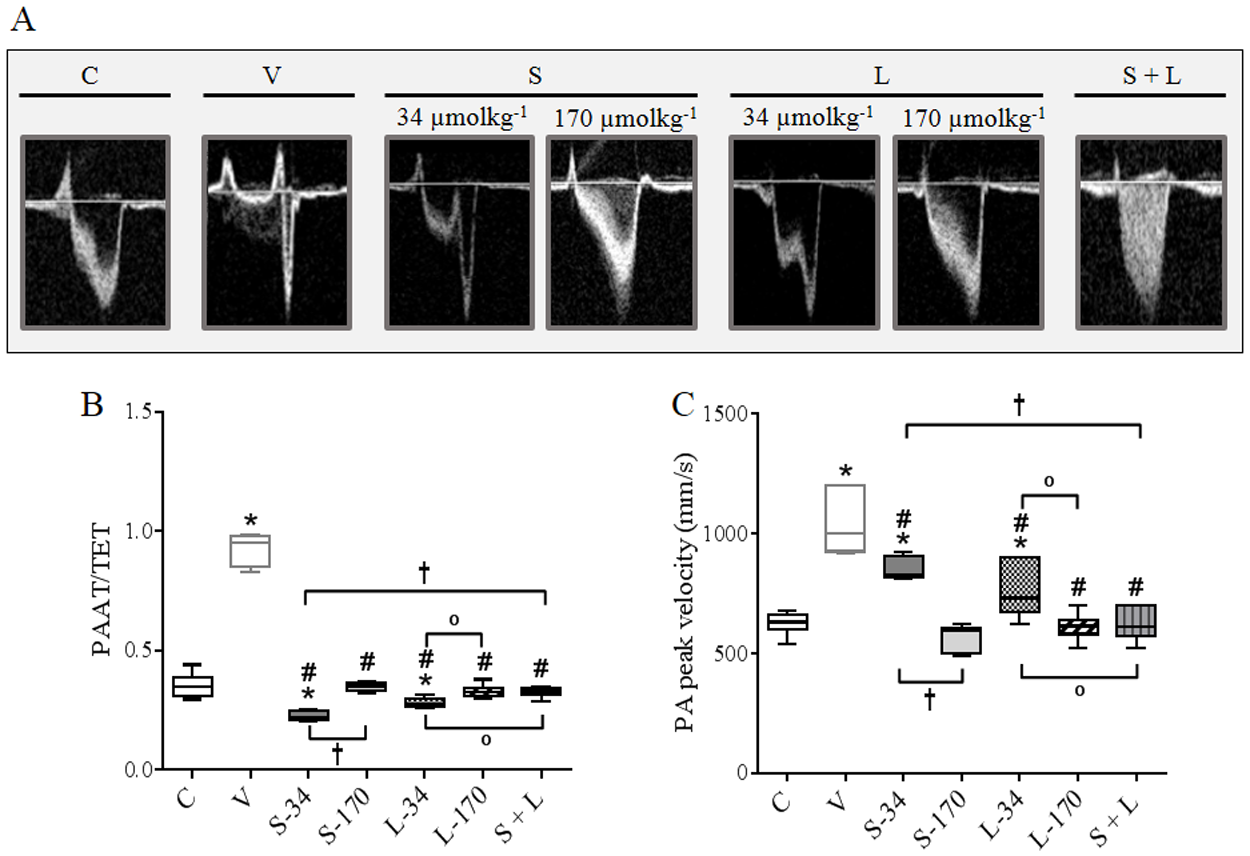
Effects of MCT injection and treatments with vehicle, monotherapy, or combination of sildenafil and LASSBio-1359 on the PA outflow profile. **(a)** Representative images of the PA outflow profile in the group receiving MCT injection (after 28 days). **(b)** Ratio between PA acceleration time and TET. **(c)** PA peak velocity. Data represent the mean ± SEM (n = 6 rats per group). One-way analysis of variance was used to determine the significance of differences among groups. Significance of interactions between groups was determined by Tukey’s post-hoc tests. *P < 0.05 compared with C group rats; *#P* < 0.05 compared with V group rats; †*P* < 0.05 compared with S-34 group rats, and °*P* < 0.05 compared with L-34 group rats. C, control; V, MCT + vehicle; S-34, MCT + sildenafil (34 μmolkg^-1^day^-1^); S-170, MCT + sildenafil (170 μmolkg^-1^day^-1^); L-34, MCT + LASSBio-1359 (34 μmolkg^-1^day^-1^); L-170, MCT + LASSBio-1359 (170 μmolkg^-1^day^-1^); S + L, combination of sildenafil and LASSBio-1359 (34 μmolkg^-1^day^-1^ of each drug); PA, pulmonary artery; PAAT, pulmonary artery acceleration time; TET, total ejection time of the right ventricle; MCT, monocrotaline.

At the end of the protocol, Doppler imaging echocardiographic data indicated improvement of PA flow in MCT-injected rats that were treated with a high-dose monotherapy (170 μmolkg^-1^day^-1^) of sildenafil or LASSBio-1359. It was observed recovery of the ratio between PA acceleration time and RV total ejection time (TET; Fig 3B), and of the PA peak velocity (Fig 3C). Fourteen days of treatment with sildenafil or LASSBio-1359 alone at a dose of 34 μmolkg^-1^day^-1^ only partially reversed the deleterious effect of MCT on PA flow (Fig 3A, 3B and 3C). Note that combined treatment with both drugs (34 μmolkg^-1^day^-1^ each) significantly reversed the PA flow impairment in rats with PH in comparison to the V, S-34, and L-34 groups (Fig 3A, 3B and 3C).

### Effects of treatments on pulmonary vascular function and RV hemodynamics

**Fig 4:**
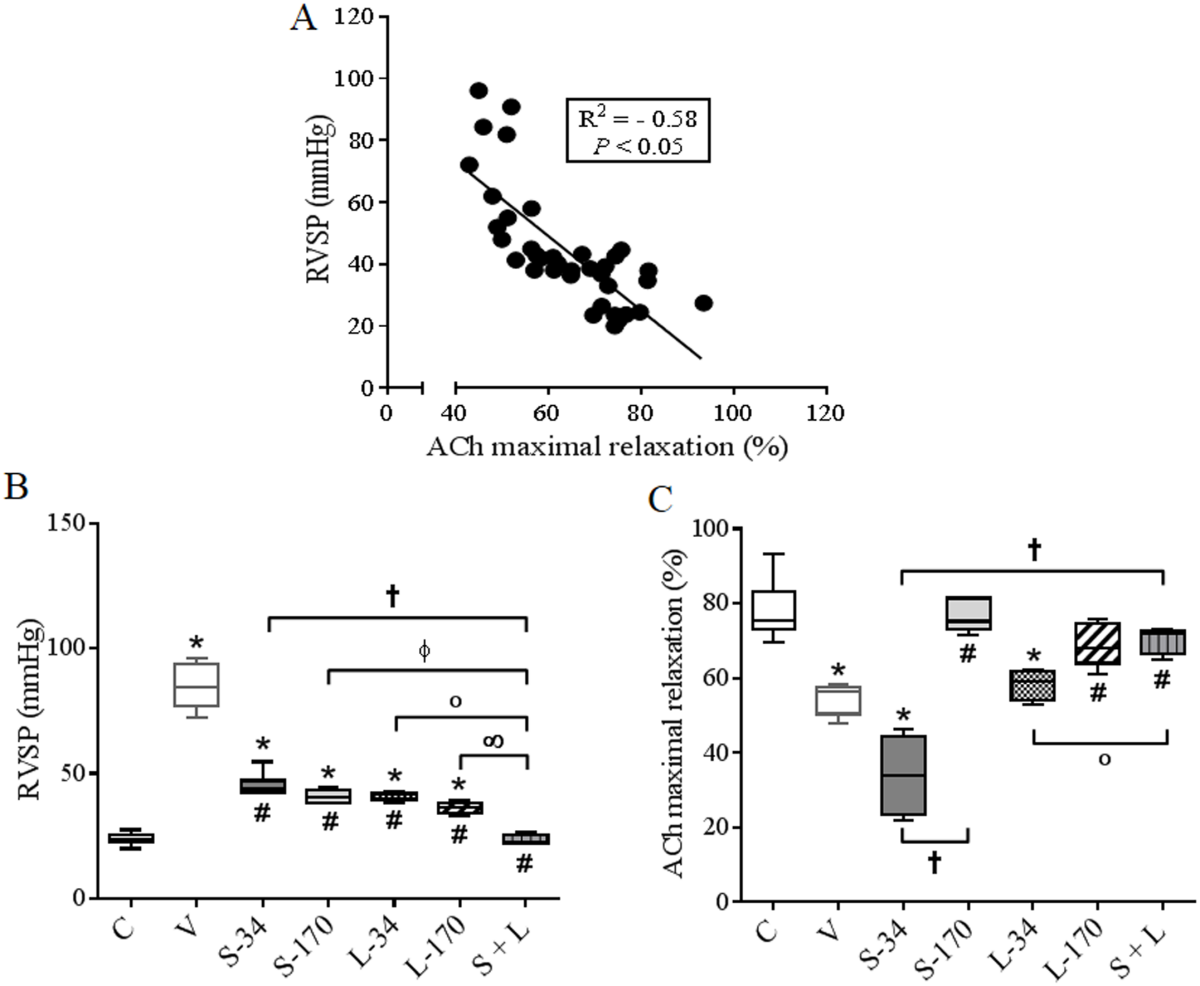
Effects of MCT injection and treatments with vehicle, monotherapy, or combination of sildenafil and LASSBio-1359 on PA reactivity to ACh and RVSP on day 28. **(a)** Linear regression between RVSP and ACh-induced maximal relaxation in PAs from experimental groups. **(b)** ACh-induced maximal relaxation. **(c)** RVSP. Data represent the mean ± SEM (n = 6 rats per group). One-way analysis of variance was used to determine the significance of differences among groups. Significance of interactions between groups was determined by Tukey’s post-hoc tests. *P < 0.05 compared with C group rats; *#P* < 0.05 compared with V group rats; †*P* < 0.05 compared with S-34 group rats, °*P* < 0.05 compared with L-34 group rats, Φ*P* < 0.05 compared with S-170 group rats, and ∞*P* < 0.05 compared with L-170 group rats. C, control; V, MCT + vehicle; S-34, MCT + sildenafil (34 μmolkg^-1^day^-1^); S-170, MCT + sildenafil (170 μmolkg^-1^day^-1^); L-34, MCT + LASSBio-1359 (34 μmolkg^-1^day^-1^); L-170, MCT + LASSBio-1359 (170 μmolkg^-1^day^-1^); S + L, combination of sildenafil and LASSBio-1359 (34 μmolkg^-1^day^-1^ of each drug); ACh, acetylcholine; RVSP, right ventricular systolic pressure; MCT, monocrotaline.

Fig 4A shows that ACh-induced PA relaxation is significantly correlated with RVSP. As expected, MCT reduced ACh efficacy 28 days after the beginning of the protocol. This pulmonary endothelial dysfunction may partially account for the higher RVSP in the V group compared to the C group (Fig 4B and 4C). Low-dose monotherapy (34 μmolkg^-1^day^-1^) with sildenafil or LASSBio-1359 did not change these alterations in pulmonary vascular reactivity and RVSP in rats that developed PH (Fig 4B and 4C). High-dose monotherapy (170 μmolkg^-1^day^-1^) with sildenafil or LASSBio-1359 reversed the pulmonary endothelial dysfunction (Fig 4B). However, these high-dose treatments only partially reduced RVSP in rats with MCT-induced PH compared to the V group (Fig 4C). The combination of sildenafil and LASSBio-1359 at 34 μmolkg^-1^day^-1^ for each drug was the only therapeutic treatment that completely reversed the changes in PA function and RV hemodynamics in rats with PH.

### Effects of monotherapy and combination therapy with sildenafil and LASSBio-1359 on RV geometry and function

**Fig 5:**
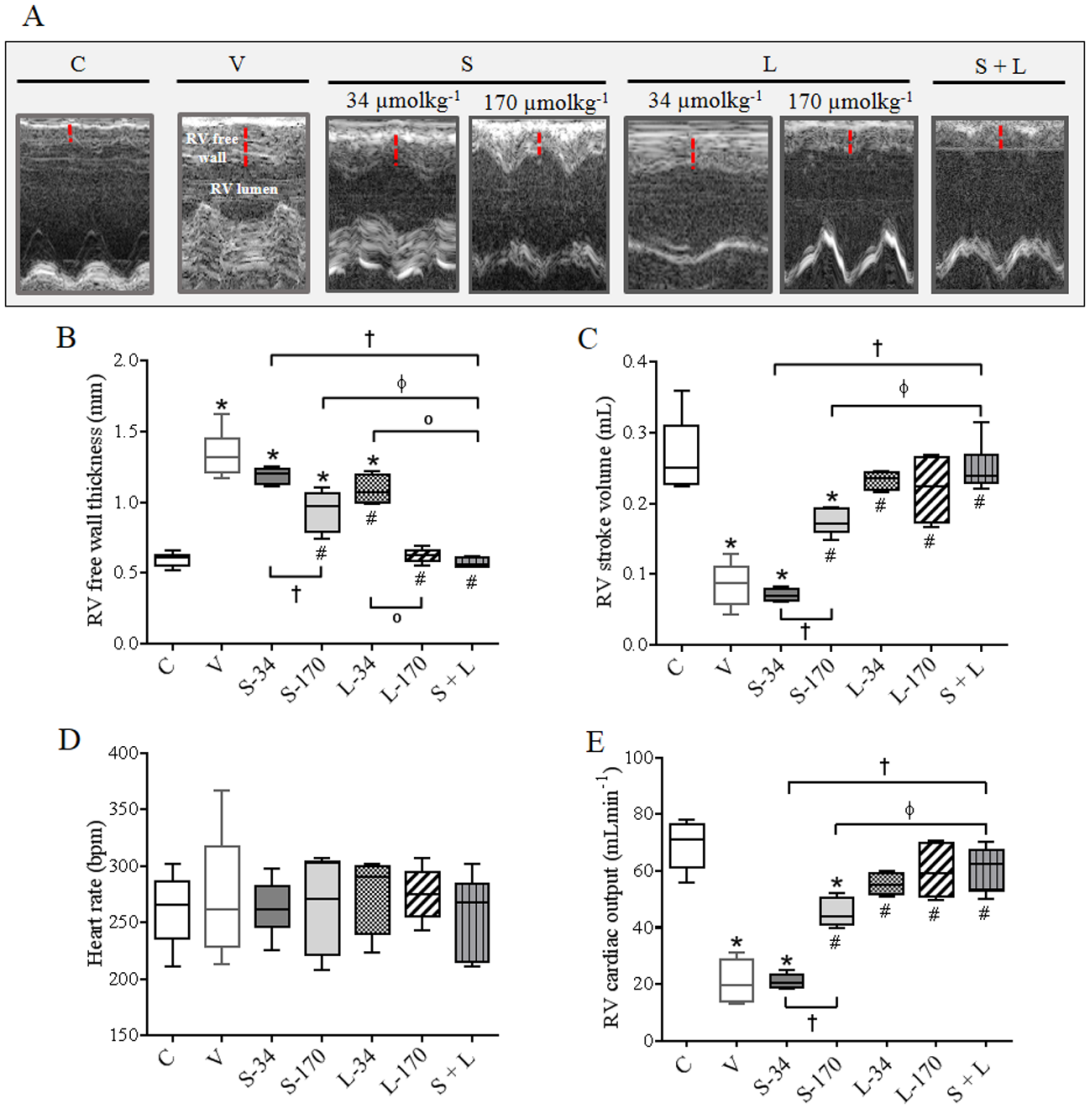
Effects of MCT injection and treatments with vehicle, monotherapy, or combination of sildenafil and LASSBio-1359 on RV geometry and function 28 days after MCT administration. **(a)** Representative images of RV free wall obtained by M-mode echocardiography. **(b)** RV free wall thickness. **(c)** RV stroke volume. **(d)** Heart rate. **(f)** RV cardiac output. Data represent the mean ± SEM (n = 6 rats per group). One-way analysis of variance was used to determine the significance of differences among groups. Significance of interactions between groups was determined by Tukey’s post-hoc tests. *P < 0.05 compared with C group rats; *#P* < 0.05 compared with V group rats; †*P* < 0.05 compared with S-34 group rats, °*P* < 0.05 compared with L-34 group rats, and Φ*P* < 0.05 compared with S-170 group rats. C, control; V, MCT + vehicle; S-34, MCT + sildenafil (34 μmolkg^-1^day^-1^); S-170, MCT + sildenafil (170 μmolkg^-1^day^-1^); L-34, MCT + LASSBio-1359 (34 μmolkg^-1^day^-1^); L-170, MCT + LASSBio-1359 (170 μmolkg^-1^day^-1^); S + L, combination of sildenafil and LASSBio-1359 (34 μmolkg^-1^day^-1^ of each drug); RV, right ventricle; MCT, monocrotaline.

Fig 5A shows M-mode echocardiographic images from all experimental groups and Fig 5B shows the RV free wall thickness. At the end of the experimental protocol, the long-term RV overload induced an increase in RV wall thickness in MCT-injected rats (Fig 5B). This alteration in RV geometry was responsible for the reduction in RV cardiac output (Fig 5E) in PH rats, which was calculated by combining the RV stroke volume (Fig 5C) and heart rate (Fig 5D). Monotherapy with sildenafil did not reverse RV free wall hypertrophy and RV dysfunction at a dose of 34 μmolkg^-1^day^-1^, and it partially normalized these RV alterations at 170 μmolkg^-1^day^-1^, as indicated by the higher RV free wall thickness, lower RV stroke volume, and lower cardiac output in the S-170 group compared to C group rats. Low-dose monotherapy with LASSBio-1359 (34 μmolkg^-1^day^-1^) partially reduced RV wall thickness (Fig 5B) and significantly normalized RV function in MCT-challenged rats (Fig 5C and 5E, respectively).

Administration of 170 μmolkg^-1^day^-1^ of LASSBio-1359 significantly reversed RV hypertrophy and dysfunction. Echocardiographic data show that the combination of sildenafil with LASSBio-1359 at a dose of 34 μmolkg^-1^day^-1^ for each drug significantly reduced the RV free wall hypertrophy in rats with MCT-induced PH in comparison to the V, S-34, S-170, and L-34 groups (Fig 5B, 5C and 5E). RV systolic function in rats with MCT-induced PH that were treated with a combination of low-dose (34 μmolkg^-1^day^-1^) sildenafil and LASSBio-1359 for 14 days was similar to that of the C group (Fig 5C and 5E). As shown in Fig 5D, heart rate did not differ among the groups.

### Effects of treatments on survival

**Fig 6:**
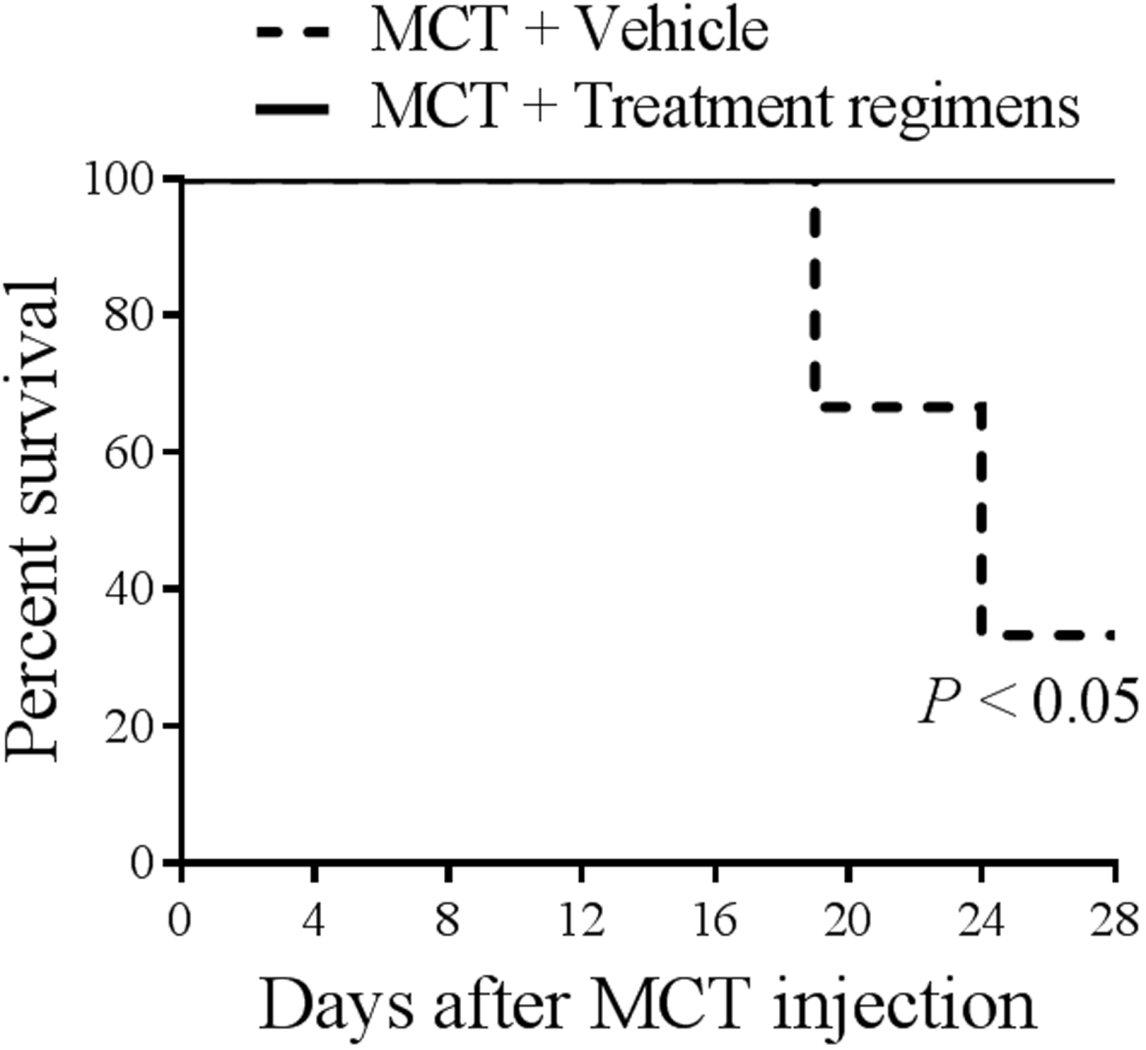
Kaplan—Meier survival curve of MCT-injected rats treated with vehicle (DMSO) or therapeutic regimens. MCT, monocrotaline.

A Kaplan—Meier survival curve showed significant difference in survival rate between MCT-injected rats treated with vehicle or therapeutic regimens (Fig 6). As we observe, PH animals that received vehicle treatment had a significant reduction in the percent survival. Importantly, there were no deaths in any of the treatment groups from day 14 to 28 of protocol.

## Discussion

The main finding of this study indicated that the combination of orally administered sildenafil and LASSBio-1359 at low doses reversed the functional cardiopulmonary system changes, in rats submitted to MCT-induced PH. In contrast, low-dose monotherapy with sildenafil or LASSBio-1359 was not effective in treating MCT-induced PH. Additionally, high dose of LASSBio-1359 has a nearly identical therapeutic effect as the low dose of combination therapy in most of our experiments, an important finding for reducing the probability of side effects. These results suggest a synergistically interaction between sildenafil and LASSBio-1359 individually working in separates target of action.

The deleterious effect of PH involves a sequence of mechanisms that affect pulmonary vascular and heart tissue. Persistent and excessive proliferation of pulmonary vascular smooth muscle and endothelial cells combined with abnormal vasoconstriction and increased collagen deposition in small resistance pulmonary vessels may lead to chronic elevation of RV afterload and myocardial injury, with subsequent heart dysfunction, failure, and premature death [23, 24]. Thus, treatment strategies for PH patients must consider this complex process. According to disease severity, therapies should utilize pharmacological substances with multiple mechanisms of action for safety and cardiovascular benefits.

Several clinical studies have shown that combination therapy is beneficial for PH patients [25–29]. Nevertheless, most randomized controlled trials showing these benefits have adopted treatment regimens with high dosages or progressive increases in combined drug amounts to reach better outcomes than monotherapy [4, 28–31]. Accordingly, it is important to consider that if either an initial (upfront) or a sequential combination strategy is selected, then an evaluation of the safety profiles of the drugs is needed to minimize potential toxicity and side effects.

In clinical practice, combining drugs with synergistic effects is important as it allows the use of smaller amounts of the constituent drugs to treat disease. Understanding drug synergism may also help guide new drug development, reduce toxic interactions, and provide new directions for experimental designs [14]. In our *in vitro* experiments, the isobolographic analyses revealed a significant difference between the experimental EC_50_ and theoretical EC_50_ values described previously [16]. We demonstrated the synergistic effect between sildenafil and LASSBio-1359 *in vitro* when the combination of both substances produced a significant PA relaxation when compared to the single dose. This finding indicates that LASSBio-1359 may increase the pharmacological activity of sildenafil. These data reinforce the synergistic interaction observed with sildenafil and LASSBio-1359 in an experimental model of erectile dysfunction [12].

The classical mechanisms of actions of both drugs studied here in inducing cardiovascular effects are well described. We have recently reviewed the benefits and roles of the A_2A_R in the cardiopulmonary system and its potential as a target for the treatment of PH in the future [32]. Inhibition of PDE5 is also salutary for the regulation of the pulmonary vascular bed physiology [33, 34].

Understanding the synergism between sildenafil and LASSBio-1359 may be useful in elucidating a pathway to induce relaxation in the pulmonary vasculature of PH patients and in developing new theories to treat PH in the future. Tallarida (2007) described the properties of two combined substances that act on multiple receptors, and reported numerous mechanisms that may explain why the action of sildenafil is enhanced in the presence of LASSBio-1359. For instance, LASSBio-1359 may enhance the affinity of sildenafil for its receptor by causing the release of some novel effect-enhancing chemical or by promoting delivery of sildenafil to its receptor [16].

After observing the *in vitro* synergism between sildenafil and LASSBio-1359 in PAs from Wistar rats, we evaluated the synergistic effects of these drugs in an *in vivo* model of PH. The *in vivo* protocol to induce PH in male Wistar rats involved the administration of MCT, a mitogenic alkaloid that causes pathological abnormalities. These abnormalities differ from those of PH observed in humans, due to presence of plexiform lesions in the pulmonary vasculature. However, the MCT model induces intense RV hypertrophy and dysfunction [9, 11, 20, 21, 35–37], which are the major determinants of human life expectancy in clinical practice, making it a suitable animal model of PH.

The pulsed-wave doppler echocardiographic findings revealed that from 14 to 28 days after MCT injection, PH rats had alterations in the PA flow, probably due to hypertrophy and stiffness of the vessel. Normalization of blood flow in PH subjects may reduce RV injury and promote a better outcome [38].

Importantly, 14 days of combined therapy with sildenafil and LASSBio-1359 offered more benefits than either compound alone in reducing MCT-induced PA flow changes. The combined therapy prevented the formation of a sharp peak at early systole. Moreover, the recovery of PAAT/TET ratio and PA peak velocity were clearly observed when we administered both compounds in combination reinforcing the *in vitro* synergism.

MCT causes chronic inflammation of the lung tissue, which damages the vascular endothelium and leads to dysfunction. As a result, there is decreased production of NO by endothelial cells of pulmonary arterioles. The decreased bioavailability of NO reduces its antiproliferative effects and thus contributes to enhanced pulmonary vascular resistance [9, 11, 39]. These events lead to increased pulmonary arterial pressure and pulmonary vascular remodeling. Here, we showed that the increased RVSP, measured 28 days after MCT application, was significantly correlated with alterations in reactivity to ACh in the PA endothelium. Surprisingly, the combined modality with sildenafil and LASSBio-1359 synergically reversed the endothelial dysfunction in MCT-treated rats. It was the only treatment that completely reduced the RV pressure overload in the PH rats, and the RVSP in the S + L group was similar to that of C group.

Echocardiography also showed that MCT-induced PH was responsible for changes in RV geometry and function. Animals in the V group developed intense RV wall hypertrophy, in part through adaptive myocardial remodeling by day 28 of the protocol. Concomitant RV dysfunction and failure were reflected by the reduced RV stroke volume and cardiac output. Importantly, simultaneous treatment with low doses of both sildenafil and LASSBio-1359 in rats with PH demonstrated synergistic actions on RV structure and function. This combination of a PDE5i with an A_2A_R agonist abolished all of the RV alterations measured by echocardiography. In contrast, low-dose treatment of rats with MCT-induced PH with either sildenafil or LASSBio-1359 alone only partially reversed or had no beneficial effects on RV. The combination of both compounds also reduced postmortem heart and RV weights, which corroborated our echocardiographic results; however, single treatments with either sildenafil or LASSBio-1359 alone at low doses did not produce these effects.

A rational strategy for developing new therapies to treat PH and its detrimental effects on heart muscles, especially RV structure and physiology, should extend beyond antiremodeling and vasodilation effects on the pulmonary vasculature, as most currently available drugs do [40, 41]. Besides having beneficial effects on lung vessels, new therapies should have multiple actions on the whole cardiopulmonary system. In this study, we evaluated the potential effects of sildenafil and LASSBio-1359 on the cardiopulmonary system of rats with PH. Because both substances synergically act through different pathways involved in PH pathophysiology, we propose that this drug combination has a novel and dual mechanism of action that is particularly important in counteracting the adverse effects of PH on pulmonary circulation and RV function and structure.

The synergism of sildenafil and LASSBio-1359 may also confer cardioprotection by inhibiting maladaptive RV remodeling and hypertrophy through well-described molecular mechanisms. PDE5 inhibition by sildenafil suppressed chamber and myocyte hypertrophy and improved *in vivo* heart function in an animal model of chronic pressure overload [42]. Together with the activation of A_2A_R in cardiac fibroblasts, accumulation of cAMP, and reduction of collagen synthesis [43], the dual mechanisms promoted by the combined administration of sildenafil with LASSBio-1359 would explain the favorable reduction in RV free wall hypertrophy observed in our M-mode echocardiography.

LASSBio-1359 has a lower *in vitro* affinity (micromolar) for the A_2A_R when compared to the widely studied ligand of this adenosine receptor CGS 21680 (nanomolar) [11]. Nevertheless, LASSBio-1359 showed a satisfactory *in vivo* efficacy in the cardiopulmonary system of rats with PH through oral administration, with no side effects in the systemic circulation [11]. CGS 21680 has a small oral bioavailability (Webb et al., 1992), what justify its restricted administration by parenteral routs in most of the studies that evaluated its *in vivo* effects [44–47]. Furthermore, CGS 21680 is a potent systemic vasodilator [44, 45], what may represent a limiting pharmacological characteristic of this compound for the treatment of PH. According to findings of our research group (data not shown), LASSBio-1359 is a selective agonist of the A_2A_R with lack of additional affinity for other cardiopulmonary targets, i.e., PDE5. Additionally, administration of different therapeutic regimens improved survival in MCT-induced PH rats by the end of experimental protocol. This explains our hypothesis of combining LASSBio-1359 with a PDE5i in the future to promote synergistic and beneficial responses in the small circulation of PH subjects.

While PH is more common among women [48], we have studied here the synergism between an A_2A_R agonist and a PDE5i in male rats in order to examine the pharmacologic potential of this new therapeutic modality independent of sex. Indeed, future studies are needed to examine its role in female rodents with and without endogenous oestradiol, a cardioprotective hormone [49].

In conclusion, our results show that there is *in vitro* activation of the A_2A_R by LASSBio-1359 in PAs from Wistar rats. Moreover, we found that LASSBio-1359 has a synergistic effect with the PDE5i, sildenafil, which may provide a successful new strategy to treat PH. The subsequent *in vivo* combination of both drugs, at doses that did not show effectiveness in monotherapy, was able to reverse the unfavorable effects of MCT-induced PH on the cardiopulmonary system of male rats. The combination treatment attenuated endothelial dysfunction in the PAs, which in turn reduced RV overload and myocardial stress. Furthermore, this synergic combination may have a cardioprotective effect on the RV of rats with MCT-induced PH because it reversed RV hypertrophy and dysfunction. These results indicate that combined treatment with LASSBio-1359 and sildenafil at low doses may reduce side effects and increase efficacy in PH patients who do not respond to sildenafil monotherapy.
